# Metabolomic Profiling in Renal Cell Carcinoma Patients: News and Views

**DOI:** 10.3390/cancers13205229

**Published:** 2021-10-18

**Authors:** Gaetano Aurilio, Matteo Santoni, Francesco Massari, Alessia Cimadamore, Alessandro Rizzo, Veronica Mollica, Elena Verri, Nicola Battelli, Rodolfo Montironi

**Affiliations:** 1 Medical Oncology Division of Urogenital and Head & Neck Cancer, IEO European Institute of Oncology IRCCS, 20141 Milan, Italy; elena.verri@ieo.it; 2 Oncology Unit, Macerata Hospital, 62100 Macerata, Italy; mattymo@alice.it (M.S.); nicola.battelli@sanita.marche.it (N.B.); 3 Medical Oncology, IRCCS Azienda Ospedaliero-Universitaria di Bologna, Via Albertoni-15, 40138 Bologna, Italy; francesco.massari@aosp.bo.it (F.M.); alessandro.rizzo11@studio.unibo.it (A.R.); veronica.mollica2@unibo.it (V.M.); 4 Section of Pathological Anatomy, School of Medicine, Polytechnic University of the Marche Region, United Hospitals, 60126 Ancona, Italy; a.cimadamore@staff.univpm.it (A.C.); r.montironi@staff.univpm.it (R.M.)

**Keywords:** renal cell carcinoma, histology, metabolomic profiling, therapeutics

## Abstract

**Simple Summary:**

Understanding the metabolic basis of renal cell carcinoma (RCC) has been of paramount importance in defining therapeutic management in clinical practice. Unfortunately, cancer drug resistance continues to be a major cause of treatment failure. Accordingly, developing new treatment perspectives targeting new metabolisms can contribute to overcoming the development of multidrug resistance, and thus optimize patient cure. In this review, we will define and discuss the outline of RCC metabolism, and we will describe certain therapeutic strategies targeting metabolic pathways. The PI3K/Akt signaling pathway continues to be the main target of clinical investigation in RCC patients. Data from metabolic pathways such as c-Met, GSH, and HDAC, variously targeted in combination with PI3K/Akt inhibitors, seem to offer new potential treatment opportunities for the research community. In this view, further studies are warranted.

**Abstract:**

Background: We address novelty regarding metabolomic profiling in renal cell carcinoma (RCC) patients, in an attempt to postulate potential treatment strategies. Methods: A large-scale literature search in existing scientific websites focusing on the keywords “renal cell carcinoma”, “clear cell histology”, “papillary histology”, “metabolomic profiling”, and “therapeutics” was performed. *Results*: The PI3K/Akt signaling pathway is key in clear cell RCC metabolism and accordingly several drugs are presently available for routine use in clinical practice. Along this line, new treatment combinations against PI3K/Akt family members are currently under clinical investigation. On the other hand, new developed targets such as c-Met tyrosine kinase domain, glutathione (GSH) metabolism, and histone deacetylases enzymes (HDAC), as well as therapeutic strategies targeting them are currently being tested in clinical trials and here discussed. Conclusions: In RCC patients, the PI3K/Akt signaling is still the most effective targetable pathway. Targeting other metabolic pathways such as c-Met, GSH, and HDAC appears to be a promising approach and deserve further insights.

## 1. Background

Renal cell carcinoma (RCC) is highly dependent on dysregulated expression of several genes, and the greater understanding of metabolomic RCC profiling has played a key role in optimizing the therapeutic management of patients in clinical practice. Histopathological classification of RCC distinguishes three most frequent subtypes: clear cell RCC (ccRCC), which accounts for the 70–75% of all RCC; papillary RCC (10–15%); and chromophobe RCC (5%) [1]. The phosphatidylinositol-3 kinase (PI3K)/protein kinase B (Akt) signaling has been considered as notorious in the main critical pathway for carcinogenesis in RCC, especially for ccRCC histology. The serine/threonine kinase Akt is the most relevant downstream member of this pathway, having a crucial transduction role throughout the signaling cascade [2]. Until now, it was estimated that at least 17 genes were strictly correlated with RCC development, and alterations of these genes have been suggested to elicit a dysregulation of various cellular metabolisms involved in oxygen, energy, and nutrient control. Data on cancer cell metabolism have shown that cancer cells are prone to a metabolic energy reprogramming for supporting cell growth and division, and such metabolism reprogramming has been listed as one of the cornerstones of cancer development [3]. Along this line, the Warburg effect—otherwise known as aerobic glycolysis—is one of the first metabolic alterations to be detected, and is present in the majority of cancer cells. It displays the attitude of tumor cells to prefer glycolysis in place of oxidative phosphorylation (OXPHOS), even when oxygen is available [4], a process maximally expressed in ccRCC [5]. Epigenetic mechanisms have also been recognized for deregulating certain metabolic processes implicated in growth and cell survival of RCC [6].

Despite recent immune-based treatment combinations [7,8,9] successfully enlarging the therapeutic landscape of patients with mRCC, cancer drug resistance continues to be a major cause of treatment failure. Accordingly, developing new treatment perspectives targeting new metabolisms can contribute to overcoming the development of multidrug resistance.

In the present article, we aimed to address novelty regarding metabolomic profiling in patients with RCC, in an attempt to postulate potential therapeutic strategies.

## 2. Outline of Renal Cell Carcinoma Metabolism

### 2.1. Metabolic Reprogramming

Glucose metabolism is of key importance in cancer development. Glycolysis is a metabolic pathway that converts glucose into pyruvate, finally generating energy to sustain cellular growth. Under normal oxygen condition, glucose is metabolized in pyruvate, and the latter produces the molecule acetyl-coenzyme A, which fuels the OXPHOS pathway. The entire process culminates into the synthesis of adenosine triphosphate (ATP), the most energetic compound for almost all metabolic reactions. In this scenario of normoxia, lactate production is minimal and basal rate of glycolysis occurs; conversely, under low oxygen conditions, glycolysis is facilitated, and pyruvate is converted into lactate [10].

Cancer cells benefit from different oxygen levels due to a well-known chaotic tumor vasculature. However, regardless of this, cancer cells display a highly aerobic glucose consumption, a key feature of cancer metabolism known as aerobic glycolysis, or the Warburg effect. Data from Warburg and coworkers demonstrated that cancer cells required more glucose than healthy cells, in a ratio of approximately 10 to 1, and originated a greater production of lactate [4]. This occurred through both increased glycolysis and with a sufficient oxygen supply.

**Figure 1:**
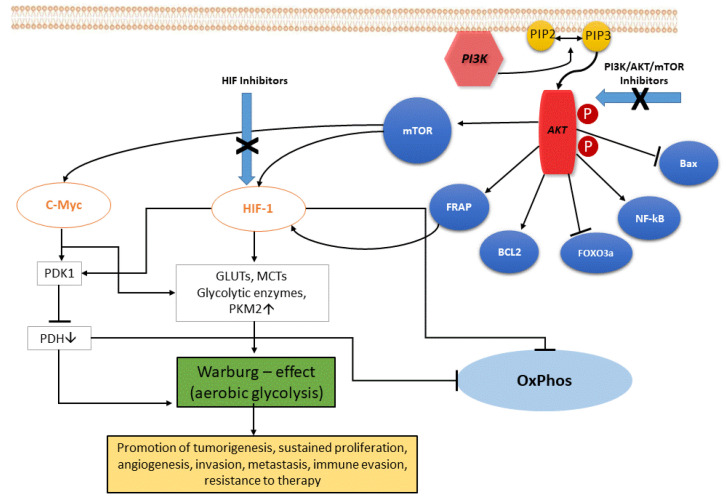
The PI3K/Akt signaling pathway. PI3K, phosphatidylinositol 3 kinase; Akt, protein kinase HIF, hypoxia-induced factor; FoxO3a, Forkhead box O3; mTOR, mammalian target of rapamycin; FRAP, FKBP-rapamycin associated protein; BCL2, B-cell lymphoma 2; Bax, Bcl-2-associated X protein; eNOS, endothelial cell nitric oxide synthase; cMyc, cellular Myc oncogene; HIF, hypoxia-inducible factor; GLUTs, glucose transporters; MCTs, monocarboxylate transporters; PKM2, pyruvate kinase muscle isozyme M2; PDH, pyruvate dehydrogenase (complex); PDK1, pyruvate dehydrogenase kinase 1; OxPhos, oxidative phosphorylation.

In ccRCC, the upregulation of the PI3K/Akt pathway is the major cause of the Warburg effect. The autophosphorylation of PI3K induces Akt activation through PIP2-to-PIP3 conversion: the Akt-mediated signaling cascade leads to phosphorylation of target downstream effectors (genes and proteins) able to promote the Warburg effect, and therefore a wide range of cellular functions, as illustrated in Figure 1. Akt activation induces stimulation of Bcl-2 and inhibition of Bax, helping cancer cell survival and accordingly their resistance [11]. Akt overexpression also inactivates certain FOX-family members that play a role in mechanisms of drug resistance. In addition, Akt is able to overactivate the NF-kB system, which in turn fuels the growth of cancer cells, thus promoting drug resistance [12]. Akt signaling activates the hypoxia-inducible transcription factors (HIF) family, thus increasing the transcription of glucose transporters, such as GLUT, and of most glycolytic enzymes, such as PDK and PKM2, finally promoting aerobic glycolytic metabolism, and leading to increased lactate production and OXPHOS inactivity.

As a consequence, some advantages for cancer cells do occur. Firstly, lipid synthesis, as well as amino acid and nucleotide production, are sustained through the glycolysis process, which supports cell growth in a condition of oxygen lability. Additionally, the Warburg phenomenon decreases reactive oxygen species (ROS) levels by inducing antioxidant glutathione, thus helping cells against oxidative stress. Lastly, increased lactate production via glycolysis generates an acidic tumor microenvironment, which has been correlated with aggressive cancer phenotype, specifically a high risk for recurrences, as well as occurrence of metastases [13].

In the tumor microenvironment, lactate induces HIF-1-alpha stabilization, and hence cancer and endothelial cells respectively exhibit hyperexpression of vascular endothelial growth factor (VEGF) and its type-2 receptor (VEGFR2) [14]. Accordingly, cancer invasion, migration, angiogenesis, and overall growth and cell proliferation are sustained. Of interest, high lactate expression in tumor microenvironment reduces the activity of T lymphocytes and natural killer cells, as well as cytokine release by dendritic cells, overall displaying an immunosuppressive action [15]. Therefore, lactate was thus configured as a metabolic cancer enhancer. Hence, the angiogenic and glycolytic phenotypes in ccRCC are strictly connected to one another, and often coexist in the same tumor. RCC has been defined as a “metabolic disease” for its characteristic metabolic defects and alterations that are consequences of the unique genetic background that drive this cancer [16].

### 2.2. Epigenetic Alterations

Epigenetic alterations are chromatin impairments that cause reversible modifications in gene expression sequences. In normal tissue, epigenetic mechanisms are able to provide genomic balance, as well as chromosome regulation.

Contrarily, along with several other factors, metabolic boosts can induce abnormal epigenetic changes that may contribute to triggering a malignant transformation. An altered epigenetic regulation is a distinctive trait of cancer growth, and encompasses both proto-oncogene activation and onco-suppressor gene downregulation. It can be sustained by certain mechanisms, such as DNA methylation, histone modifications, and chromatin remodeling, briefly illustrated here as follows. The first one is a key, well-investigated epigenetic mechanism. Cancer cells exhibit extensively hypomethylated DNA, which is responsible for genomic instability, the latter characterized by high mutational burden, proto-oncogene induction, and loss of genomic imprinting. An abnormal hypermethylated status instead occurs for gene promoters, and correlates with reduced transcription expression of onco-suppressor genes. The second one deals with post-translational changes of histone tails, mainly as methylation and acetylation of specific amino acid sequences. These processes are regulated by modification-inducing enzymes called histone methyltransferases and histone acetyltransferases, respectively, and by enzymes removing such modifications, also called lisine demethylases and histone deacetylases (HDAC), respectively. The third one regards chromatin remodeling complexes, particularly through inactivating mutations occurring in polybromo-1 (PBRM1) and BRCA-associated protein-1 (BAP1) genes. PBRM1 is a chromatin regulating gene that modulates the accessibility to DNA transcription, and data support PBRM1 inactivation as a promoter for cell proliferation and migration. BAP1 is a tumor suppressor gene and coregulates many biological mechanisms, from cell cycle function to cell death, passing through metabolic processes and DNA regulation. RCC with BAP1 deficiency is significantly associated with decreased survival [17]. Therefore, taking into account all of the above, abnormal DNA methylation, histone post-translational changes, and chromatin remodeling complexes are the epigenetic signature of RCC, prone to control transcription and DNA integrity via chromatin modifications, and thus culminating in increased carcinogenesis and tumor aggressiveness.

## 3. Therapeutic Strategies Targeting Metabolic Pathways

### 3.1. PI3K/Akt Pathway

**Figure 2:**
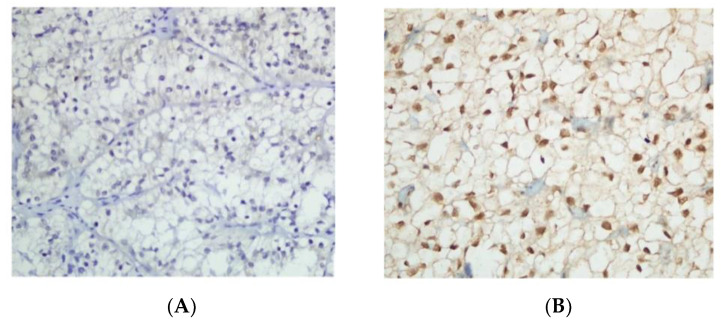
Clear cell renal cell carcinoma with negative HIF-2α expression (**A**) and positive HIF-1α expression (**B**) (×400). Reproduced under the terms of the Creative Commons Attribution 4.0 International License (http://creativecommons.org/licenses/by/4.0/, accessed on 18 October 2021) (reference: https://doi.org/10.1186/s13000-018-0742-8).

The PI3K/Akt pathway is notoriously key in ccRCC metabolism, as previously mentioned. Several therapeutics targeting this signaling pathway have been developed, respectively directed toward the tyrosine kinase (TK) domain of the VEGF receptor (cabozantinib, axitinib, pazopanib, lenvatinib), or dual inhibitors toward the VEGF and PDGF receptors (sunitinib, sorafenib), as well as the monoclonal antibody anti-VEGF (bevacizumab) and two PI3K/AKT/mTOR inhibitors (everolimus, temsirolimus). These drugs—in other words, metabolic agents—have significantly improved patient survival outcomes. Currently, new small molecules that inhibit the linkage between HIF-2α and HIF-1β are under clinical investigation, especially PT2399, a selective HIF-2 antagonist, based on the preclinical evidence that PT2399 affects cell replication and growth of both ccRCC cell lines and xenograft models [18]. Figure 2 displays ccRCC with negative HIF-2α expression (A) and positive HIF-1α expression (B). Of interest, phase I data from a first-in-class HIF-2α antagonist, such as PT2385, are available [19]. A total of 51 ccRCC patients with advanced disease and heavily pretreated with a median of four prior therapies, including at least one VEGF inhibitor, were treated with PT2385. No dose-limiting toxicity emerged, and no treatment discontinuation due to adverse events was observed; in addition, 66% of patients had a disease control rate (DCR), mainly as a stable disease [19] (Table 1). Along this line of research, very recently, preliminary efficacy data from a two-cohort phase II study of the oral hypoxia-inducible factor 2α (HIF-2α) inhibitor MK-6482, in combination with cabozantinib in metastatic ccRCC patients, were presented at the last virtual 2021 Genitourinary Cancers Symposium. The findings showed a DCR in 92% of patients and a median PFS of 16.8 months; there were grade 3 treatment-related adverse events in ≥5% of patients, while no grade 4 toxicity or deaths occurred (Table 1).

### 3.2. c-Met Tyrosine Kinase Domain

c-Met is the TK receptor for hepatocyte growth factor, involved in cancer cell proliferation, VEGF-driven angiogenesis, and tumor metastasis. The expression of c-Met was proved to be prominent in sarcomatoid and papillary type 1 RCC, but also in advanced stage and low-grade differentiation tumors. Immunohistochemical staining for c-Met is also common in collecting duct carcinoma, as well as in urothelial carcinoma of the renal pelvis. Literature data suggest that c-Met expression is unfavorably associated with many survival endpoints [20]. Activating c-Met gene mutations or amplifications have been reported in papillary RCC patients, and c-Met-driven RCC tumors may benefit from c-Met TK inhibitor treatment. In a phase II study, the potent oral multikinase inhibitor foretinib has been explored in papillary metastatic RCC (mRCC) tumors. A total of 74 patients who received no more than one prior systemic therapy were stratified according to c-Met mutational burden. Patients with a germline c-Met mutation had a more favorable tumor response than those not mutated, while c-Met amplification did not correlate with tumor response. The safety profile of foretinib was characterized by manageable toxicities, and an overall response rate equal to 13.5% among mutated patients was observed [21]. The EORTC 90101 CREATE phase II trial investigated the small molecule c-Met TK inhibitor crizotinib in papillary mRCC patients with or without c-Met mutations. According to the results of this trial, c-Met mutated patients exhibited a higher ORR than nonmutated ones (50% versus 6.3%, respectively). Moreover, the experimental treatment was well tolerated, and provided long-lasting tumor control in patients with c-Met mutations, although some responses were also noted in nonmutated patients or in patients with unknown c-Met status, suggesting the action of other alterations of c-Met, as well as alternative pathways [22]. In a phase II biomarker-based study, the highly selective c-Met TK inhibitor savolitinib demonstrated 18% of partial responses in patients with c-Met-driven papillary mRCC tumors [23]. Along this promising line, the efficacy of savolitinib was tested versus the standard comparator sunitinib in the SAVOIR phase III, open-label, randomized trial, in which only patients with c-Met-driven (centrally confirmed) papillary mRCC tumors were enrolled. From a total of 254 screened patients, 60 of these received savolitinib or sunitinib; although survival data, ORR, and safety profile were numerically in favor of savolitinib versus sunitinib, and no statistically significant difference was reported between the two arms [24] (Table 1). Further insights into savolitinib in MET-driven papillary RCC tumors may be useful to clarify its role as a putative treatment option.

### 3.3. GSH Metabolism

Increased accumulation of ROS is a frequent condition in several cancer types. ROS play a crucial role in cell metabolism, since moderate levels of ROS promote tumor growth and metastasis by stimulating oncogenic signaling pathways. In this regard, glutathione (GSH) metabolism primarily acts to scavenge intracellular ROS in an attempt to maintain a balanced oxidative stress, and thus protect cancer from apoptosis.

Cystine/glutamate transporter xCT expression is promoted in response to ROS-stimulating agents, namely sodium arsenite and hydrogen peroxide, leading to increased GSH production. Indeed, xCT regulates the uptake of cysteine, an important element for the biosynthesis of GSH. From this, targeting xCT is becoming a potential therapeutic opportunity within GSH metabolism. Sorafenib, a multiple TK inhibitor in RCC tumors, was recently proved to have a role against xCT, causing reduced cysteine uptake, ROS accumulation, and GSH exhaustion [25]. Of interest, sulfasalazine, an xCT inhibitor drug historically used for intestinal inflammation, demonstrated anticancer activity in VHL-deficient RCC tumors [26].

Glutaminase 1 (GLS1) in turn is a crucial mitochondrial enzyme that converts glutamine into glutamate within the GSH metabolism. Targeting GLS1 substantially contributes to destroying GSH metabolism, thus affecting redox homeostasis and ATP synthesis. The oral selective GLS1 inhibitor CB-839 has determined efficacy in RCC, offering opportunities to combine it with other known therapeutics [27]. In particular, the combination of CB-839 and everolimus has demonstrated a synergistic anticancer propensity in both in vitro RCC cell lines and in vivo RCC models.

Glutamate–cysteine ligase (GCL) is another key enzyme within the GSH metabolism, converting glutamate into γ-glutamyl cysteine, and contributing to GSH synthesis and thus to the preservation of GSH homeostasis. Buthionine sulfoximine (BSO) is a potent and specific inhibitor of GCL, and there is evidence that BSO has a synergistic effect when combined with anthracyclines, melphalan, and other cytotoxic drugs in multiple myeloma, neuroblastoma, and breast cancer [28,29]. In mRCC patients, resistance to sorafenib has been linked to increased HIF induction. From this, since BSO can affect HIF production, combining sorafenib with BSO would be rational in order to revert sorafenib resistance in mRCC patients.

Lastly, encouraging clinical activity and toxicity data of CB-839 in combination with cabozantinib in heavily pretreated mRCC patients, mainly with cc histology, were presented at the 2019 Genitourinary Cancer Symposium (Table 1).

### 3.4. HDAC Inibition

The HDAC inhibitor abexinostat, in combination with the anti-VEGF pazopanib, has been used in 51 patients with solid tumors in a phase I trial encompassing a dose expansion in mRCC patients. More than half of patients (n = 30) had received ≥1 VEGF-targeting therapy, and 10 patients had a prior progression under pazopanib. Overall, median duration of response was equal to 9 months, and of relevance, 70% of pazopanib-refractory patients had a lasting tumor remission. Further, toxicity by using the combination was well manageable [30] (Table 1). Trichostatin A (TSA), another HDAC inhibitor, has demonstrated potent antitumor activity in breast cancer cell lines, both in vitro and in vivo [31]. Sato and colleagues conducted a metabolome and transcriptome analysis in human RCC cell lines to assess whether TSA-induced changes in gene profiling and metabolic regulation had a role in sunitinib resistance. The results of this study indicated that adding TSA to sunitinib-refractory RCC cells produced significant inhibition of cancer growth, inducing cell death by PARP cleavage without affecting TK activity [32]. In addition, preclinical evidence pointed out synergistic anticancer activity between HDAC and mTOR inhibitors. Notwithstanding, when matching the mTOR inhibitor everolimus with the HDAC inhibitor panobinostat in a dose-escalation phase I trial, in 21 ccRCC patients, disappointing results emerged, with this combination failing to improve clinical outcomes [33].

## 4. Discussion

Improvements in understanding the tumorigenesis process, along with cancer cell biology, have led to the identification of a novel cancer feature known as metabolic energy reprogramming. This is a crucial metabolic feature of RCC, maximally expressed by the Warburg effect, an almost universal process in cancer. This biological effect exerts a predominant action in RCC development by inducing the production of metabolites, which in turn contribute to regulate epigenetic factors. It is estimated that aerobic glycolysis produces only two molecules of ATP per glucose molecule, much less compared to the 36 molecules generated by the OXPHOS pathway [3]. This apparent “inefficiency” remains a matter of debate. One hypothesis holds that the Warburg effect is an advantageous process for cancer cells, since it is a faster modality to gain ATP [34]. Another theory holds that this switch inducing an acidification of the tumor microenvironment, as previously mentioned, confers meaningful advantages to cancer cells [35]. A molecular characterization of ccRCC tumors, published in *Nature* in 2013, underlined that patients with an advanced disease stage and high cell dedifferentiation were prone to a metabolic shift (Warburg effect) for energy production [6]. Along with a greater understanding of the Warburg phenomenon, as well as of the PI3K/Akt pathway, new putative therapeutic targets for glycolysis are now in focus, such as GLUT1, lactate dehydrogenase A, aldolase A, and sodium/glucose cotransporter 2 [36].

Literature data supported PI3K/Akt hyperactivation as a major contributor to developing drug resistance [37].

It is known that various cancers can exhibit different gene profiling, as well as that the same histologic subtype can genetically differ among patients, and even within a single subject, the same tumor can display a broad spectrum of genetic heterogeneity. Again, tumor heterogeneity is able to promote tumor metabolic deregulation both of enzymes and biomolecules. According to all these points, the occurrence of various metabolic phenotypes can be stimulated, and hence therapeutic strategies based on one drug are rendered insufficient. As described above, GSH metabolism regulation is key to maintaining redox homeostasis in RCC tumors. Altering this metabolism can significantly trigger RCC progression, but at the same time, targeting GSH levels can be a promising treatment strategy. In this regard, affecting GSH as an unique target has been proven as not useful enough to kill RCC cell lines; consequently, combination strategies targeting independent cell regulatory mechanisms are being developed. Following promising data regarding a 100% DCR with the combination of selective GLS1 inhibitor CB-839 and everolimus in a phase I trial in ccRCC patients [38], a randomized, double-blind, placebo-controlled phase II trial of everolimus with or without CB-839 in advanced or mRCC patients has recently completed its accrual, and the results are awaited ([NCT 03163667). Further, CB-839 combined with nivolumab is under investigation in a phase I/II study, and preliminary results underline a 74% DCR in RCC subjects with clear cell histology (NCT02771626). Again, CB-839 added to poly(ADP-ribose) polymerase (PARP) inhibitors in vitro and in vivo in VHL-deficient ccRCC tumors displayed cell growth inhibition [39]. Accordingly, a phase Ib/II study of CB-839 plus the PARP inhibitor talazoparib in patients with advanced/metastatic solid tumors, among which was ccRCC, was recently terminated [NCT03875313].

As previously mentioned, HDAC inhibition plays a pivotal role in the field of epigenetic alterations, in particular within the mechanism of histone modifications. Anti-HDAC agents have mainly been investigated in patients with blood malignancies, while there is paucity of studies in nonblood tumors. Data from Aggarwal and colleagues resulting from testing of the combination of the HDAC inhibitor abexinostat and pazopanib [30] in a phase I trial of patients with solid tumors were challenging, as the first clinical evidence of an epigenetic modulation by using HDAC inhibition to reverse tumor resistance to VEGF inhibitors was shown. Along this line, the restoration of TK activity in sunitinib-resistant RCC cell lines by adding the HDAC inhibitor TSA showed promising preclinical evidence [32].

Although a two-drug combination treatment appears to be a more effective strategy in mRCC patients, phase I data with the oral HIF-2α antagonist PT2385 as a single agent in heavily pretreated (range: one to seven prior therapies) mccRCC patients were encouraging. Indeed, PT2385 was overall well tolerated and showed DCR in 66% of patients, no treatment discontinuation due to toxicity occurred, and more than half of the patients enrolled in the expansion phase had lasting therapy for ≥1 year. The authors finally stated that the study agent showed promising efficacy with an acceptable safety profile [19]. From this, over the mRCC course and following many lines of treatment, it cannot be excluded that a certain percentage of mRCC patients, most likely a minority, may benefit from such a treatment approach as monotherapy for further disease control.

## 5. Conclusions

Several therapeutic strategies targeting different metabolic pathways in RCC have been developed in the last decades. The TK inhibitors sunitinib and pazopanib developed first for such disease have maintained a role in the treatment landscape of metastatic patients, and the metabolic-oriented research focusing on HDAC inhibition indisputably confirms a certain scientific interest. Along this line, a remarkable tentative result for increasing the performance of the more novel approved TK inhibitor cabozantinib comes from recent preliminary data in mccRCC patients in which the addition of an HIF-2α inhibitor to cabozantinib improved clinical outcomes (2021 Genitourinary Cancer Symposium).

Further understanding of the metabolic pathways and their modification in cancer cells offers the chance to develop new therapies directed toward specific targets. Survival outcomes of mRCC patients are a continuous clinical priority, and developing new treatment perspectives targeting different cancer metabolisms can contribute to overcoming drug resistance, and thus optimize patient cure.

## Figures and Tables

**Table 1:** New molecules/combinations targeting metabolic pathways in metastatic RCC patients.

Pathways Involved	Description	Phase	Size	Findings [Ref.]
HIF-2α antagonist PT 2385	PT2385 PO BID 100–1.800 mg doses	I	51	66% DCR (mainly SD) No DLT [19]
	1–7 prior therapies			
	Histology: cc			
HIF-2α antagonist MK-6482 and VEGFA inhibitor Cabozantinib	MK-6482 120 mg PO + Cabozantinib 60 mg PO QD	II	52	92% DCR mPFS: 16.8 mo No G4 toxicity or deaths
	<2 prior therapies			
	Histology: cc			
MET/VEGFR2 inhibitor Foretinib (F)	F (A) 240 mg PO 1–5 days q14; F (B) 80 mg PO QD	II	74	13% ORR among MET mutated mPFS: 9 mo Manageable toxicity [21]
	<1 prior therapy			
	Histology: papillary			
MET inhibitor Crizotinib	Crizotinib 250 mg PO BID	II	23	ORR: 50% vs 6% 1 y PFS: 75% vs 27% 1 y OS: 75% vs 71% Acceptable toxicity [22]
	Histology: papillary			
	MET+ and MET- subcohorts pts			
MET inhibitor Savolitinib and VEGF inhibitor Sunitinib	Savolitinib 600 mg PO QD vs Sunitinib 50 mg PO QD 4:2 ws	III	60	mPFS: 7 mo vs 5 mo (*p* = 0.31) G3 toxicity: 42% vs. 81% [24]
	MET+			
	Histology: papillary			
GLS-1 inhibitor-GSH linked and VEGFA inhibitor Cabozantinb	CB-839 (GLS-1 inhibitor) 600–800 mg PO BID + Cabozantinib 60 PO QD	I	13	ccRCC: 50% ORR, 100% DCR Acceptable toxicity
	Histology: cc and papillary permitted			
	≥1 prior anti-VEGF therapy			
HDAC inhibitor Abexinostat and VEGF inhibitor Pazopanib	Abexinostat PO BID 1–5, 8–12, 15–19 days (A) or 1–4, 8–11, 15–18 days (B) + Pazopanib PO QD	I	51	mDOR: 9 mo
				Pazo-refractory pts: lasting response [30]

Abbreviations: RCC, renal cell carcinoma; P, phase; N, size; Ref., reference; PO, per oral; BID, twice daily; cc, clear cell; DCR, disease control rate; SD, stable disease; DLT, dose limiting toxicity; QD, once daily; mPFS, median progression-free survival; mo, months; G4, grade 4; (A), cohort A; q14, every 14 days; (B), cohort B; ORR, objective response rate; OS, overall survival; 4:2 ws, 4 weeks on and 2 weeks off; DCR, disease control rate; mDOR, median duration of response; pts, patients.
